# A Novel Technique for Laparoscopic Salvage of CAPD Catheter Malfunction and Migration: The Santosh-PGI Hanging Loop Technique

**DOI:** 10.1155/2015/684976

**Published:** 2015-03-30

**Authors:** Santosh Kumar, Shivanshu Singh, Aditya Prakash Sharma, Manish Rathi

**Affiliations:** ^1^Department of Urology, Postgraduate Institute of Medical Education and Research, Chandigarh 160012, India; ^2^Department of Nephrology, Postgraduate Institute of Medical Education and Research, Chandigarh 160012, India

## Abstract

CAPD catheter malfunction is a common problem. Obstruction due to wrapping by appendices epiploicae of sigmoid colon has been rarely reported in literature. We report a case of CAPD catheter malfunction caused by catheter tip migration and obstruction by appendices epiploicae that was successfully managed by laparoscopic hanging loop technique. This case report highlights the ease with which epiplopexy can be performed and catheter tip migration can be prevented by this innovative laparoscopic procedure.

## 1. Introduction 

CAPD (Continuous Ambulatory Peritoneal Dialysis) is a prevalent mode of renal replacement therapy with nearly 197,000 ESRD patients using it globally [1]. One of the important aspects of CAPD catheter is its adequate function defined as one that allows adequate inflow and/or outflow of dialysate solution [2]. CAPD catheter malfunction is one of the major causes for its discontinuation. Catheter malfunction can result from its luminal occlusion due to omental or small bowel wrapping, malposition, or migration of catheter. Obstruction due to appendices epiploicae of the sigmoid colon is a rarely reported etiology [3]. Laparoscopic salvage of malfunctioning catheters helps in reducing patient's morbidity and need of intermediary hemodialysis. However, recurrent malfunction had motivated surgeons to innovate newer methods of laparoscopic CAPD catheter salvage. We report a case of CAPD catheter occlusion caused by wrapping of appendices epiploicae of sigmoid colon, a rare cause. Laparoscopic “Santosh-PGI Hanging Loop Technique,” an innovative concept with multiple benefits, salvaged it.

## 2. Case Report

**Figure 1:**
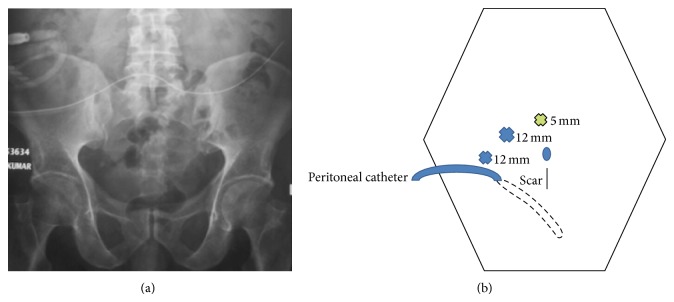
X-ray abdomen showing migrated CAPD catheter lying outside the pelvis (a). Illustration of laparoscopic port placement (b). The middle 12 mm blue cross is the camera port while the corners 12 mm and 5 mm are the working ports.

A 54-year-old gentleman having diabetes and hypertension was diagnosed as end-stage renal disease requiring renal replacement therapy. He opted for CAPD. His serum creatinine was 7.9 mg/dL. Straight Tenckhoff CAPD catheter insertion was done by standard open technique. Patient was discharged on postoperative day three. Two weeks after insertion, CAPD initiation was planned. However, there was slow inflow and a trickling outflow. Diagnosis of CAPD catheter malfunction was made. X-ray abdomen revealed malposition of CAPD catheter (Figure 1(a)). It was lying outside the pelvis in left lateral abdomen. Initial management with laxatives, bowel enema, failed with no improvement in dialysate flux. Repositioning of CAPD catheter was attempted under fluoroscopic guidance but it was unsuccessful. Keeping a diagnosis of CAPD catheter malposition with suspected omental wrapping, decision was made for diagnostic laparoscopy along with a salvage procedure as required.

**Figure 2:**
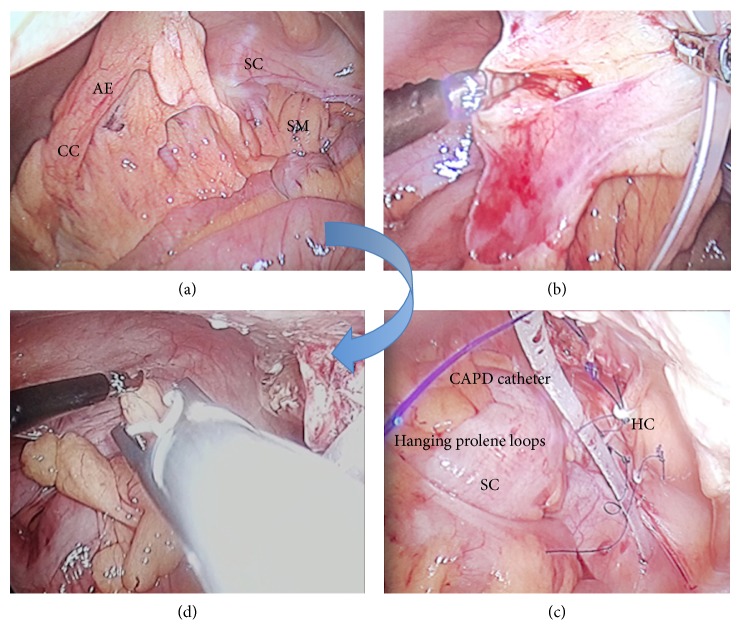
Laparoscopic view demonstrating wrapped-up CAPD catheter by appendices epiploicae of sigmoid colon (a). Dissection of the catheter from epiploicae using harmonic shear device (b). CAPD catheter hanging freely over prolene loops that are fixed to the pelvic parietal peritoneum using hemostatic clips (c). Epiplopexy using hemostatic clips (d) (AE = appendices epiploicae; CC = CAPD catheter; SC = sigmoid colon; SM = sigmoid mesocolon; HC = hemostatic clips).

Under general anesthesia, laparoscopy was performed with the patient in supine position with Trendelenburg tilt. Three ports were placed including one 12 mm camera and two working ports (Figure 1(b)). To our surprise, the catheter was engulfed by appendices epiploicae of sigmoid colon (Figure 2). With the help of laparoscopic harmonic shear device, the catheter was completely dissected off from the appendices epiploicae. It was repositioned in the pelvis. Further, in an attempt to prevent recurrent CAPD catheter occlusion and migration, an innovative “Santosh-PGI Hanging Loop Technique” was applied. In this, prolene 2-0 needleless suture was taken. Two knots were applied on it, one on each side. This suture was then passed into peritoneal cavity through one of the ports. A loop was made around the CAPD catheter and was fixed to the pelvic peritoneum with help of hemostatic clips. Similarly, two more loops were applied. In this way, the CAPD catheter was hanged freely underneath the pelvic peritoneum with the help of prolene suture. To prevent appendices epiploicae from falling back on the catheter, they were fixed to the left lower abdominal parietal peritoneum using hemostatic clips. On table catheter patency and position were confirmed. Postoperative period was uneventful. Low volume dialysis was started on 1st postoperative day. Normal volume dialysis was resumed by day 9. At 6-month follow-up his catheter is functioning well. Repeat X-ray imaging revealed its pelvic position.

## 3. Discussion

From 1997 to 2008, the number of peritoneal dialysis patients in developing countries increased by 24.9 patients per million population while in developed countries it increased by 21.8 per million population [1]. CAPD catheter malfunction is one of the major causes for discontinuation of this therapeutic modality. Incidence ranges from 12 to 73% [4]. Catheter malfunction accounts for 20% of patient transfers to hemodialysis [5]. Malfunction can result from occlusion of catheter by omental or small bowel wrapping with adhesions, fibrin clot, migration or malposition, and tunnel infections. Various series have reported omental wrapping and malposition to be the most common cause of malfunction [2, 6, 7]. Appendices epiploicae has been rarely reported as a cause of catheter occlusion [3]. CAPD catheter salvage instead of removal helps in functional rescue, thereby prolonging catheter life, decreasing patient morbidity, reducing financial burden, and allowing early institution of peritoneal dialysis, thereby decreasing dependence on intermediary hemodialysis [8]. Laparoscopic salvage of CAPD catheters has several reported advantages [8]. Laparoscopy provides a direct vision of the pathology, helps in diagnosis of other intra-abdominal pathologies, has lower incidence of postsurgical adhesions, incision related complications, postprocedure pain, thereby resulting in early ambulation, shorter hospital stay with quicker return to work. It allows early institution of peritoneal dialysis and better functional survival of catheter. Salvage procedures include laparoscopic unwrapping of omentum with omentectomy, omentopexy, [2] adhesiolysis, or laparoscopic milking of occluded catheters [9]. Laparoscopically salvaged catheters have been shown to have reasonably good period of catheter survival with a median of 163 days in some studies [7]. Initial success rates of as high as 100% have been reported [8] highlighting the usefulness of salvage procedures.

Catheter tip migration contributes to a substantial proportion of catheter malfunction. Those not corrected by conservative or stiff wire manipulation require surgical intervention. Laparoscopic salvage has definite advantages over open surgical procedures. Laparoscopic preperitoneal placement and pelvic fixation using extracorporeal knotting have been reported in an attempt to prevent catheter migration [10]. We report an innovative, easily performed technique of laparoscopic rescue of CAPD catheter malfunction due to wrapping by appendices epiploicae of sigmoid colon and catheter tip migration. In this, we first unwrapped the catheter from appendices epiploicae. The catheter was freely hanged underneath the pelvic peritoneum over prolene suture loops that were fixed to the peritoneum with hemostatic clips using laparoscopic clip applicator. The tip of the catheter was in the pelvic cavity. This technique has several advantages. Suture fixation is easy with hemostatic clips. The catheter hangs freely in the loop thereby allowing easy removal or exchange in the future. No separate incisions are required for subcutaneous suture fixation as it is done intraperitoneally. In order to prevent appendices from falling back on catheter, it was fixed to left lateral wall with hemostatic clips. This technique highlights the simplicity with which the catheter migration and occlusion can be prevented.

## 4. Conclusion 

CAPD catheter malfunction is a common problem. Laparoscopic salvage of such catheters decreases patient's morbidity. The laparoscopic hanging loop technique with epiplopexy using hemostatic clips helps in effectively preventing catheter tip migration out of pelvic cavity and rewrapping of catheter by appendices epiploicae.
